# A gene expression estimator of intramuscular fat percentage for use in both cattle and sheep

**DOI:** 10.1186/2049-1891-5-35

**Published:** 2014-06-16

**Authors:** Bing Guo, Kritaya Kongsuwan, Paul L Greenwood, Guanghong Zhou, Wangang Zhang, Brian P Dalrymple

**Affiliations:** 1 Key Laboratory of Meat Processing and Quality Control, Synergetic Innovation Center of Food Safety and Nutrition, College of Food Science and Technology, Nanjing Agriculture University, Nanjing 210095, P.R. China; 2 CSIRO Animal, Food and Health Sciences, St. Lucia QLD 4067, Australia; 3 Now at; National Institute of Animal Health, 50/2 Kasetklang, Ladyao, Bangkok 10900, Thailand; 4 CSIRO Animal, Food and Health Sciences, Armidale NSW 2350, Australia; 5 NSW Department of primary Industries, Armidale NSW 2350, Australia

**Keywords:** Cattle, Gene expression phenotype, IMF%, Sheep

## Abstract

**Background:**

The expression of genes encoding proteins involved in triacyglyceride and fatty acid synthesis and storage in cattle muscle are correlated with intramuscular fat (IMF)%. Are the same genes also correlated with IMF% in sheep muscle, and can the same set of genes be used to estimate IMF% in both species?

**Results:**

The correlation between gene expression (microarray) and IMF% in the longissimus muscle (LM) of twenty sheep was calculated. An integrated analysis of this dataset with an equivalent cattle correlation dataset and a cattle differential expression dataset was undertaken. A total of 30 genes were identified to be strongly correlated with IMF% in both cattle and sheep. The overlap of genes was highly significant, 8 of the 13 genes in the TAG gene set and 8 of the 13 genes in the FA gene set were in the top 100 and 500 genes respectively most correlated with IMF% in sheep, *P*-value = 0. Of the 30 genes, *CIDEA*, *THRSP*, *ACSM1*, *DGAT2* and *FABP4* had the highest average rank in both species. Using the data from two small groups of Brahman cattle (control and Hormone growth promotant-treated [known to decrease IMF% in muscle]) and 22 animals in total, the utility of a direct measure and different estimators of IMF% (ultrasound and gene expression) to differentiate between the two groups were examined. Directly measured IMF% and IMF% estimated from ultrasound scanning could not discriminate between the two groups. However, using gene expression to estimate IMF% discriminated between the two groups. Increasing the number of genes used to estimate IMF% from one to five significantly increased the discrimination power; but increasing the number of genes to 15 resulted in little further improvement.

**Conclusion:**

We have demonstrated the utility of a comparative approach to identify robust estimators of IMF% in the LM in cattle and sheep. We have also demonstrated a number of approaches (potentially applicable to much smaller groups of animals than conventional methods) to using gene expression to rank animals for IMF% within a single farm/treatment, or to estimate differences in IMF% between two farms/treatments.

## Background

Consumers are prepared to pay more for meat with superior eating qualities [1]. Intramuscular fat (IMF), the flecks and streaks of fat within the lean sections of meat, which is also known as marbling, is associated with juiciness and flavour [2]. Recent research has shown that increased IMF% could dramatically improve the tenderness of lamb carcasses 5 days post-slaughter [3]. But compared to beef-related research (see [4,5]), few publications have focussed on the molecular mechanism of IMF deposition in sheep. In the past few years, only *FABP3 (H-FABP)*, *PPARG*, *DGAT1*, *LPL*, *ACACA*, *FASN (FAS)*, *FABP4*, *CPT1B* and *SCD* have been reported to directly influence IMF% status in sheep LM [6-9]. Thus, based on the limited information from sheep, it is hard to identify a set of genes to estimate IMF%.

In our previous studies in cattle, three gene sets, designated as the “TAG gene set” (triglyceride synthesis and storage), the “FA gene set” (fatty acid synthesis and storage) and the “PPARG gene set” (Peroxisome proliferator-activated receptor gamma), were identified based on the expression profiles of the genes in the LM across development in two crosses [4]. The expression of genes from these three gene sets, in particular the TAG gene set, was correlated with IMF deposition in cattle LM [4]. The TAG gene set was used to identify the effect of HGP (hormone growth promotant) treatment, site (New South Wales [NSW] and Western Australia [WA]) and Calpain/calpastatin genotype on IMF% [4].

Cattle and sheep are evolutionarily closely related [10] and are expected to exhibit many common physiological characteristics. In this study, we hypothesised that the genes in the TAG, FA and PPARG gene sets identified in cattle could also be applied to estimate IMF% in sheep. Furthermore, based on these gene sets, we evaluated the utility of single and small sets of genes to estimate IMF% in small groups of animals from both species.

## Materials and methods

Use of animals and the proce stry & Investment New South Wales (NSW) Orange Agriculture Institute Animal Ethics Committee, Commonwealth Scientific and Industrial Organisation (CSIRO) Rockhampton Animal Experimentation Ethics Committee, and the Department of Agriculture and Food, Western Australia (WA) Animal Ethics Committee.

### Sheep correlation dataset

The design of the experiment has been described previously [11]. Briefly, 20 sheep were randomly assigned to five groups, four groups of treated animals received implants containing a combination of ~42 mg trenbolone acetate (TBA) and ~4.2 mg 17-βestradiol (E2), or ~50 mg TBA alone, or ~10 mg E2 alone at the start of the trial or 20 mg oxytocin delivered by Alzet osmotic pump over 30 d at the start of the experiment and again after 30 and 60 d. Following slaughter, 50 mg of LM tissue (between the 12^th^ and 13^th^ rib) and the strip loins (6^th^ to 9^th^ rib) were collected from the right sides of the carcasses for RNA preparation and meat quality analyses, respectively. IMF% was measured in duplicate on each sample by gas chromatography (GC) as previously described [11]. Gene expression was measured using the Bovine Oligo Microarray Chip (Bovine 4x44K) from Agilent Technologies (Santa Clara CA, USA and will be described in detail elsewhere [Kongsuwan et al., in preparation]). The same platform was used for the two bovine gene expression datasets described below. The Bovine Oligo Microarray platform was used as it has a larger coverage of genes than the equivalent sheep array and using the same platform simplifies data integration and analysis.

### Cattle correlation dataset

Correlation between gene expression and IMF% in the LM muscle in a group of 48 intensively fed Brahman steers, including three tenderness genotypes, an environment contrast (growth at two different sites, New South Wales [NSW] and Western Australia [WA]) and with and without a hormone growth promotant (HGP) treatment, has been described previously [4]. IMF% was measured by Near Infrared Spectrophotometry (NIRS), duplicate measurements on single samples, as previously described [12]. Ultrasound estimation of IMF% was undertaken as previously described [13], values were the mean of five measurements.

### Cattle DE dataset

Differential expression (DE) of genes from two cattle crosses with high and medium marbling, Wagyu cross Hereford and Piedmontese cross Hereford respectively, has been described previously [4]. The cattle in this dataset were sampled at 25 mo of age whilst at pasture.

### Statistics and bioinformatics

The correlation between gene expression and IMF% was calculated using the “CORREL” function in Microsoft Excel. Student’s t-test of significance was calculated using the “TTEST” function (one tailed) in Microsoft Excel.

*P*-values for the hypergeometric distribution for a specified number of successes in a population sample were calculated using the Excel “HYPERGEOMDIST” function.

Gene enrichment analysis was undertaken by using GOrilla network tools which uses a hypergeometric statistic to quantify functional enrichment in ranked gene lists [14]. *P*-values, and the false discovery rate (FDR) *Q*-values calculated using the Benjamini and Hochberg method [15], were provided in the results output of the GOrilla website [16].

Cluster analysis was undertaken using an expectation-maximization mixture analysis algorithm (EMMIX) [17]. All three datasets were linearly rescaled to a mean of zero and a range from −0.5 to 0.5 before analysis.

The z-score normalization was used to minimise the impact of differences in levels of expression and dynamic range of expression of genes from the combed gene expression data, individual gene expression values (log2) were normalised by dividing its difference from the mean of each measurement (across the whole set or subsets of the animals) with the relevant standard deviation using Microsoft Excel.

The random sampling and calculation of mean correlation was carried out in MATLAB software R2012a using custom scripts. Random controls with 5 genes were sampled 100,000 times by using random sampling from the rescaled cattle correlation dataset, this process was repeated 10 times. Those possessing higher correlation with IMF% in cattle in each sampling process were investigated. Then correlation of average gene expression with IMF% in these random controls in sheep was calculated.

The significance of gene rankings between groups was calculated using the Mann–Whitney Test web tool [18].

Sample size determination was then performed to estimate the minimum number of animals required to significantly differentiate two groups at a *P*-value of 0.05 and confidence interval of 95% [19], A Microsoft Excel spreadsheet “LaMorte’s Power Calculator” downloaded from the web site [20] was used.

## Results and discussion

### Expression of genes in the TAG, FA and PPARG gene sets was correlated with IMF% in sheep

The correlation between gene expression and IMF% in sheep LM across the full set of 20 samples was calculated (Table 1). The 46 genes from the TAG, FA and PPARG gene sets and related genes identified in cattle were ranked in the cattle and sheep datasets by their correlation coefficients, and DE values in the cattle DE dataset (Table 1). 8 of the 13 genes in the previously defined TAG gene set were in the top 100 genes most correlated with IMF% in sheep, *P*-value = 0, 8 of the 13 genes in the previously defined FA gene set were in the top 500 genes most correlated with IMF% in sheep, *P*-value = 0, and 6 of the 15 genes in the previously defined PPARG gene set were in the top 1,000 genes most correlated with IMF% in sheep, *P*-value < 10^−10^. Five of the 25 genes most correlated with IMF% in the whole sheep dataset and 8 of top 10 genes (Additional file 1: Table S1) in the sheep TAG, FA and PPARG gene sets correlation dataset were in the TAG gene set. This result showed the applicability of the TAG gene set in sheep, higher than the FA and PPARG gene sets, and similar to the results in cattle [4]. However, some genes ranked highly in the cattle correlation and DE datasets were ranked much lower in the sheep correlation dataset. For example, *S100G* ranked 26, 61 and 4,009 in the three datasets respectively (Table 1). Whilst such large differences in ranking may reflect species differences, it is also possible that such a large difference may be due to the use of a cattle gene expression platform for sheep mRNA. Thus the utility of some of the genes in sheep requires further investigation.

**Table 1:** Rankings of genes in various datasets and average ranking

**Genes**^**1**^	**Description**	**Rank**			**Average rank**^**6**^	**Source**
		**Sheep correlation**^**3**^	**Cattle correlation**^**4**^	**Cattle DE**^**5**^		
***CIDEA***^*2*^	cell death-inducing dffa-like effector a	11	4	12	**1**	**TAG**^7^
***ACSM1***	acyl-CoA synthetase medium-chain family member 1	16	35	23	**3**	**TAG**
***ADIPOQ***	adiponectin	17	46	25	8	**TAG**
***FABP4***	fatty acid binding protein 4,adipocyte	24	54	19	**5**	**TAG**
***PLIN1***	perilipin1	25	15	49	6	**TAG**
*TUSC5*	tumor suppressor candidate 5	54	73	63	13	TAG
***LPL***	lipoprotein lipase	62	123	473	23	EMMIX A^8^
*MAL2*	mal, T-cell differentiation protein 2	67	33	177	16	FA
***PPARG***	peroxisome proliferator-activated receptor gamma	73	117	152	19	**PPARG**
***DGAT2***	diacylglycerol o-acyltransferase 2	74	6	30	**4**	**TAG**
***AGPAT2***	phosphate O-acyltransferase 2	100	21	118	14	**TAG**
***G0S2***	g0/g1switch 2	123	223	72	20	EMMIX A
***FASN***	fatty acid synthase	167	39	17	9	**FA**
***THRSP***	thyroid hormone responsive	187	13	1	**2**	**TAG**
***ELOVL6***	ELOVL fatty acid elongase 6	231	34	6	7	**FA**
*TKT*	transketolase	244	229	106	26	PPARG
*CIDEC*	cell death-inducing DFFA-like effector c	272	41	61	15	TAG
*CYB5A*	cytochrome b5 type A (microsomal)	307	64	483	27	PPARG
*BHMT2*	betaine-homocysteine S-methyltransferase 2	317	207	304	28	FA
***RBP4***	retinol binding protein 4, plasma	346	139	78	21	**FA**
***ACSS2***	acyl-CoA synthetase short-chain family member 2	440	19	43	12	**FA**
***ARSK***	arylsulfatase family, member K	447	948	502	42	**EMMIX A**
***SCD***	stearoyl-CoA desaturase	472	9	22	10	**FA**
***ACACA***	acetyl-CoA carboxylase alpha	484	56	63	17	**FA**
***ACLY***	atp citrate lyase	535	148	16	18	**PPARG**
*PLS1*	plastin1	659	502	6,513	49	TAG^9^
*TF*	transferrin	742	310	20	22	PPARG
***CPT2***	carnitine palmitoyltransferase 2	782	664	528	44	EMMIX A
*PCK1*	phosphoenolpyruvate carboxykinase 1	859	20	13	11	TAG
***PTPLB***	protein tyrosine phosphatase-like, member b	884	275	479	37	**PPARG**^**9**^
*ADIG*	adipogenin	937	183	36	24	TAG
*PCK2*	phosphoenolpyruvate carboxykinase 2 (mitochondrial)	972	92	1,629	34	FA
*INTS9*	integrator complex subunit 9	984	1,183	80	36	PPARG
*PDE3B*	phosphodiesterase 3B, cGMP-inhibited	1,426	206	1,135	40	FA
***ACER3***	alkaline ceramidase 3	1,519	683	969	52	**PPARG**
***APOA1***	apolipoprotein A-I	1,521	3,264	119	45	EMMIX A
***ARSI***	periplasmic arylsulfatase	1,865	114	7,806	43	EMMIX A
***IDH1***	isocitrate dehydrogenase 1 (NADP+), soluble	2,070	320	210	39	**PPARG**
***APOE***	apolipoprotein E	2,224	227	424	41	EMMIX A
*GSTA1*	glutathione S-transferase alpha 1	2,302	998	3	29	PPARG
***SULT1A1***	sulfotransferase family, cytosolic, 1A, phenol-preferring, member 1	2,341	3,667	172	53	EMMIX A
*ANPEP*	alanyl (membrane) aminopeptidase	2,703	2,091	87	47	FA
*HSD17B12*	hydroxysteroid (17-beta) dehydrogenase 12	2,726	74	457	35	PPARG^9^
***CLU***	clusterin	2,968	65	136	30	**FA**
***BDH1***	3-hydroxybutyrate dehydrogenase, type 1	3,240	8	1,797	31	EMMIX A
*ACSS3*	acyl-CoA synthetase short-chain family member 3	3,689	137	347	38	PPARG
***ME1***	malic enzyme 1, NADP(+)-dependent, cytosolic	3,741	69	2,457	46	EMMIX A
*S100G*	S100 calcium binding protein G	4,009	61	26	25	TAG
*INSIG1*	insulin induced gene 1	4,562	412	31	33	FA
*G6PD*	glucose-6-phosphate dehydrogenase	4,918	982	95	51	PPARG
*GPAM*	glycerol-3-phosphate acyltransferase, (mitochondrial)	5,886	1,364	185	55	TAG^9^
*FBP1*	fructose-1, 6-bisphosphatase 1	6,604	706	2	32	FA
*QPRT*	quinolinate phosphoribosyltransferase	7,565	329	137	50	PPARG
*ALAD*	aminolevulinate dehydratase	9,051	422	347	54	PPARG
*CEBPA*	CCAAT/enhancer binding protein (C/EBP), alpha	10,545	593	65	48	PPARG

^1^Full list of genes and probes is included as Additional file 1: Table S1.

^2^Genes in bold were included in the 30 gene set correlated with IMF% in cattle and sheep generated by the current study.

^3^Gene rank of the correlation coefficients in Sheep correlation dataset.

^4^Gene rank of the correlation coefficients in Cattle correlation dataset.

^5^Gene rank of DE value in Cattle DE dataset.

^6^Genes in this table were ranked from 1–55 based on their ranking in each of the cattle and the sheep correlation datasets. The average ranking across the two datasets was calculated and the genes were again ranked from 1 to 55.

^7^These genes were both included in TAG, FA and PPARG gene sets and annotated with the GO term “lipid metabolic process” and in EMMIX cluster A.

^8^Genes were annotated with the GO term “lipid metabolic process” and in EMMIX cluster A.

^9^Genes were included in each corresponding gene set in the current analysis based on the biological functions. But these genes were not the part of TAG, FA and PPARG gene sets in our previous work [4].

### Integrated analysis

The sheep correlation dataset was generated from a small group of animals, therefore it is unavoidably noisy. Hence, integrated analysis of the cattle and sheep datasets was undertaken in order to identify robust genes for use in both species.

A cluster analysis of all the genes based on their correlation coefficients in cattle and sheep and DE values in cattle was undertaken using EMMIX [17]. Each gene was assigned a clustering parameter ranging from 0 to 1, the probability for location in the two alternative clusters. The smaller group was defined as Cluster A, the other as Cluster B (Additional file 1: Table S1). Genes with positive values in all three datasets in both clusters were selected, and then submitted to GOrilla for gene ontology enrichment analysis.

Cluster B, the larger cluster, was confirmed as background because: firstly no GO terms related to lipid metabolic process were enriched in the selected subset of genes; secondly correlation coefficients and DE values of genes in the selected subset of genes were close to 0. In Cluster A, gene ontology enrichment analysis was calculated for a number of groups of genes filtered by their clustering parameter (the probability of being a member of cluster A) (Table 2). Thirty genes were in the enriched GO term, “lipid metabolic process”, with the most significant *P-*value and FDR *Q-*value in the ≥0.9 clustering parameter gene group. *P-*values and FDR *Q-*values of the groups with lower clustering parameters were progressively and dramatically reduced while only 1 or 2 new genes were added into the GO term, “lipid metabolic process” (Table 2). On the basis of this analysis, we decided to use the set of genes from ≥0.9 cut-off with the GO term “lipid metabolic process”.

**Table 2:** Gene enrichment analysis of EMMIX Cluster A

**Prob**^**1**^	**EMMIX**			**GOrrila**			
	**Genes**			***P*-value**	**FDR *Q*-value**	**Genes**^**4**^	**Enrichment**^**5**^
	**Total**^**2**^	**Positive**^**2**^	**Negative**^**3**^				
≥0.9	571	121	136	7.66E-13	8.13E-09	30	4.57
≥0.8	768	153	170	8.56E-12	9.09E-08	31	4.12
≥0.7	920	177	198	8.38E-10	2.97E-06	32	3.37
≥0.6	1109	210	237	1.79E-08	3.80E-05	33	2.93
≥0.5	1342	248	282	8.12E-08	1.08E-04	35	2.67

^1^Probability of genes to be located in cluster A.

^2^Genes with positive coordinates in all three datasets, see Additional file 1: Table S1 sheet “Genes with positive coordinates”.

^3^Genes with all negative coordinates in all the three datasets.

^4^Number of genes enriched in “lipid metabolic process” GO term from genes with all positive coordinates in all the three datasets.

^5^Enrichment (N, B, n, b) is defined as follows: N - is the total number of genes; B - is the total number of genes associated with a specific GO term; n - is the number of genes in the top of the user's input list or in the target set when appropriate; b - is the number of genes in the intersection; Enrichment = (b/n) / (B/N).

**Figure 1:**
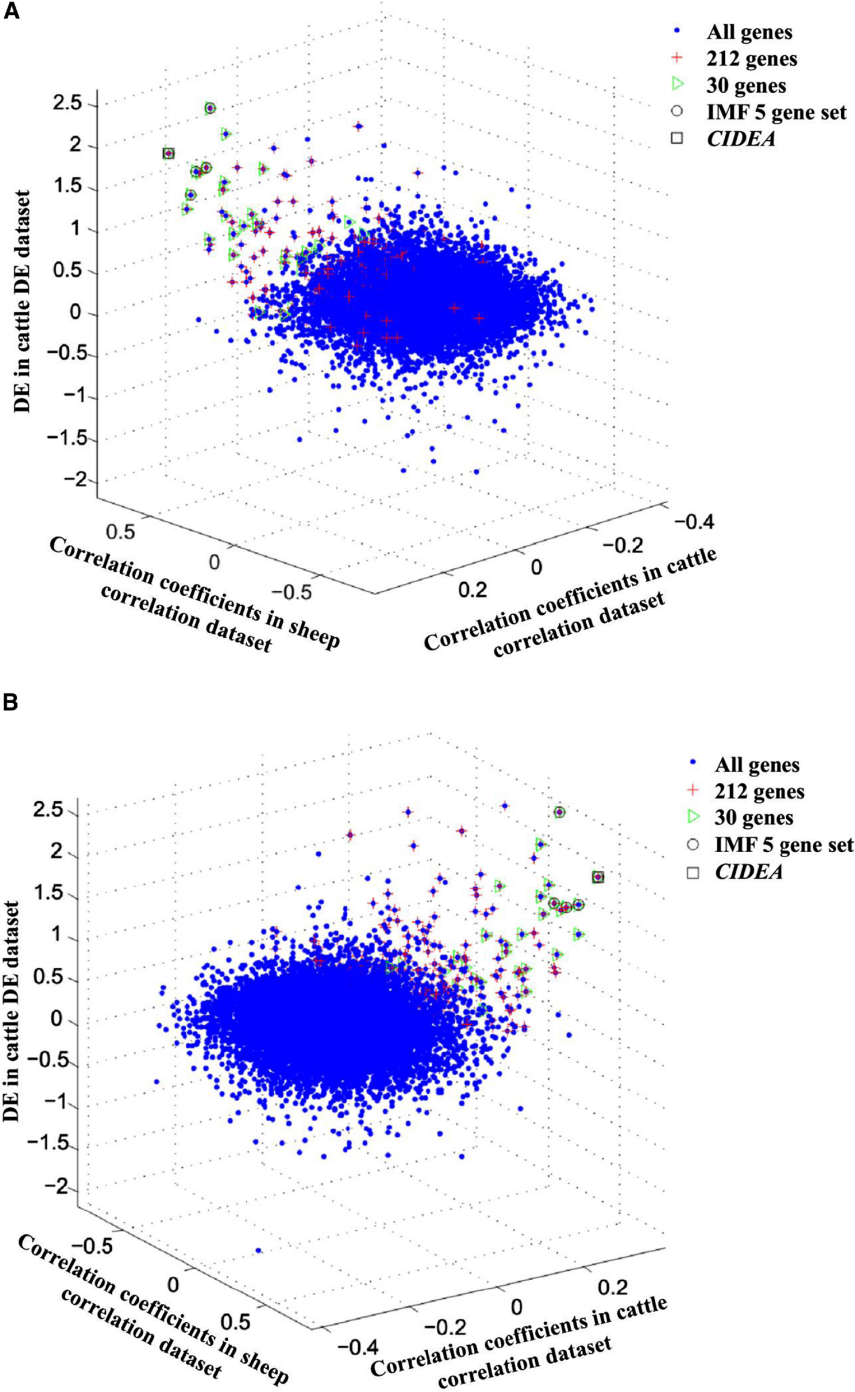
**Three dimensional plot of cattle and sheep correlation datasets and cattle DE dataset.** Data points for 13,330 probe sets with reliable data in all three datasets. The 212 genes with expression positively correlated with IMF% generated from cattle correlation and DE datasets in our previous study [4]. **A)** and **B)** are two different visual angles of this three dimensional plot.

Of 315 genes with extreme positive values in both the cattle correlation and DE datasets identified in our previous study [4], 212 were included in the integrated analysis (96 genes were lost due to poor probe performance in the sheep dataset). The set of 30 genes identified above contained 24 of these genes (Figure 1).

Ninteen genes from the previously described TAG, FA and PPARG gene sets from cattle were included in the 30 genes. The remaining 11 genes included two categories of genes. The first category included genes with a well characterised and important role in lipid metabolic process and with relatively high correlation/DE coefficients, such as *LPL* and *G0S2*[21,22]. The second category contained genes with very low correlation/DE coefficients (close to 0) and included in the lipid metabolic process GO term, such as *ARSK*, *BDH1* and S*ULT1A1*[23-25].

Of the genes reported in the literature to be important in sheep IMF deposition, *PPARG*, *LPL*, *ACACA*, *FABP4*, *FASN (FAS)* and *SCD* were included in the top 30, *FABP3 (H-FABP)*, *DGAT1*, and *CPT1B*[4,6-9] were not.

### Correlation of *CIDEA* and IMF gene set(s) with IMF% in both cattle and sheep

*CIDEA* was the highest ranked gene based on correlation coefficients in both the cattle and sheep datasets (Table 1). Thus *CIDEA* is the best candidate for use as a single gene estimator of IMF% in both species. However a single gene may not be the best estimator of IMF%. Combinations of genes may be a better solution because they will provide multiple measurements of the lipid synthesis and storage pathway thereby reducing the measurement error. The set of 30 genes described above was used to identify a gene combination(s) able to estimate IMF% equally well in both cattle and sheep. *CIDEA*, *THRSP*, *ACSM1*, *DGAT2* and *FABP4* from the TAG gene set were selected based on their combined ranking in both the cattle and sheep correlation datasets (Table 2). Consistent with our findings, two of these five genes (*DGAT2* and *FABP4*) have also been identified to be correlated with IMF in cattle previously [26,27]. We tested the top 2, top 3, top 4 and top 5 genes above (defined as the “IMF 2–5 gene sets”) to determine whether combinations of the top genes had a higher correlation with IMF% in both species than *CIDEA* alone. A simple model (average of rescaled gene expression values) was used to combine the data from the different genes.

In this study, more than 19,000 genes in cattle and more than 13,600 genes in sheep were detected by the microarray platform. Therefore, there are a very large number of possible combinations of 2–5 genes in the cattle and sheep datasets. How likely is it that a random set of 2–5 genes would be as well correlated with IMF% in both cattle and sheep as the “IMF 2–5 gene sets”? It was reasonable to test these “IMF 2–5 gene sets” based on two principles. Firstly, whether individual genes from gene combinations with higher correlation were as correlated with IMF deposition as the individual genes in the “IMF gene sets”? Secondly, whether gene combinations which exhibited a higher correlation than the “IMF 2–5 gene sets” in cattle exhibited higher correlation than the “IMF 2–5 gene sets” in sheep as well? We calculated the correlation between expression of all of the “IMF 2–5 gene sets” and IMF% and found that all the correlation coefficients were around 0.46-0.51 in both cattle and sheep (Table 3). Therefore, the IMF gene sets with 2–5 genes appeared to be equally well correlated in both cattle and sheep. For each “IMF gene set”, same size randomly selected gene combinations (defined as “random controls”) were sampled 100,000 times by using random sampling from the cattle correlation dataset. Those random controls which possessed higher correlation with IMF% than the corresponding “IMF gene sets” were recorded. Twenty six and 96 random controls containing sets of 2 and 3 genes respectively with higher correlation coefficients than the “IMF gene sets” were found in cattle. In contrast, very few random control gene sets with higher correlation coefficients were found containing 4 or 5 genes. Interestingly, most of the analysed random control gene sets with high correlation with IMF% contained at least one gene related to lipid metabolism from the set of 30 genes; the remaining genes were from other biological processes apparently unrelated to fat deposition. There were no random controls showing higher correlation with IMF% in sheep than the “IMF gene sets” with 2–5 genes. These results showed that the *P*-value for selecting a set of genes by chance which has as high correlation with IMF% in both cattle and sheep was <10^−6^, and probably very much smaller.

**Table 3:** Correlation between gene expression and IMF%

**Animals**	***CIDEA***	**IMF 5 gene set**
20 sheep	0.52	0.51
48 cattle	0.40	0.46
36 cattle^1^	0.61	0.60
NSW control group	−0.40	−0.04

^1^48 cattle excluding the NSW control group.

As previously described [4], the 48 Brahman cattle were divided into four subgroups by experimental site (NSW and WA) and treatment (with and without HGP). In the NSW control subgroup, consistent with previous analysis [4], no significantly positive correlation between expression of any of the 5 top ranking genes and IMF% was observed (Table 3 and data not shown). In addition, in this group of animals, none of the “IMF 2–5 gene sets” showed significant correlation with IMF% (Table 3 and data not shown). This is probably due to an environmental factor such as disease or nutrition. For this reason, we repeated the analysis using the remaining three subgroups of cattle (NSW HGP-treated, WA control and WA HGP-treated), 36 animals in total. Expression of *CIDEA* was now as correlated with IMF% (0.61) as the “IMF 2–5 gene sets” (0.60-0.62) when the NSW control group was not included (Table 3).

However, the group of cattle excluding the NSW control subgroup, and the sheep may not be representative of the spectrum of animals in real production systems. Thus, we repeated the analysis on all the 48 Brahman steers and 20 sheep. In the cattle, the expression of the IMF 5 gene set showed a slightly higher correlation with IMF% than *CIDEA* alone (Table 3). In sheep, the IMF 5 gene set showed similarly high correlation with IMF% as *CIDEA* alone (Table 3).

Overall, on the basis of the correlation of gene expression with IMF%, *CIDEA* alone and the IMF 5 gene set performed very similarly in sheep and in cattle and in the subsets of cattle.

### Relationship between gene expression and IMF% in both cattle and sheep

Is there a simple relationship between *CIDEA* and IMF 5 gene set gene expression and IMF% in both species facilitating the development of a gene-based test for sorting animals by their estimated IMF% in the LM?

**Figure 2:**
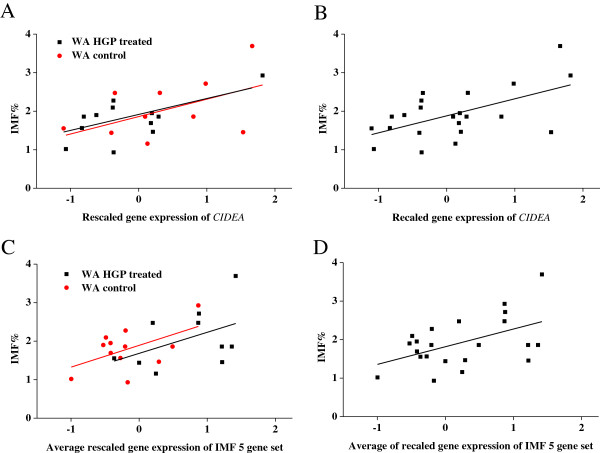
**Relationship between IMF% and gene expression in Brahman cattle. A)** IMF% and *CIDEA* expression in WA control and HGP treated animals separately. **B)** IMF% and *CIDEA* expression in WA cattle. **C)** IMF% and IMF 5 gene set expression in WA control and HGP treated animals separately. **D)** IMF% and IMF 5 gene set expression in WA cattle.

**Figure 3:**
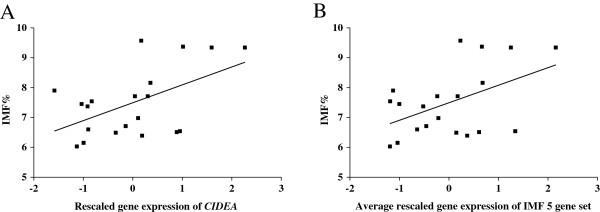
**Relationship between IMF% and gene expression in 20 sheep. A)** IMF% and *CIDEA* expression. **B)** IMF% and IMF 5 gene set expression.

The rescaled gene expression values of *CIDEA* and the IMF 5 gene set were plotted against the IMF% for cattle (Figure 2) and sheep (Figure 3). Analysing the WA control and HGP-treated animals separately demonstrated that the relationships between gene expression and IMF% were very similar (Figure 2A, C). Thus it appears that the HGP-treatment did not significantly affect the relationship between IMF% and *CIDEA*/IMF 5 gene set expression, except for the effect due to the reduced IMF% of HGP-treated animals (Figure 2C). The data from the 22 cattle (not including the NSW animals) was combined (Figure 2B, D) and similarly for the 20 sheep (Figure 3). We found similar linear relationships between IMF% with both *CIDEA* and the IMF 5 gene set (Figure 2B, D & Figure 3B) in both cattle and sheep. These results suggest that *CIDEA* and the IMF 5 gene set could be used across species and a broad range of IMF% values to estimate IMF%.

### Applications of gene estimator(s) of IMF% in both cattle and sheep

**Figure 4:**
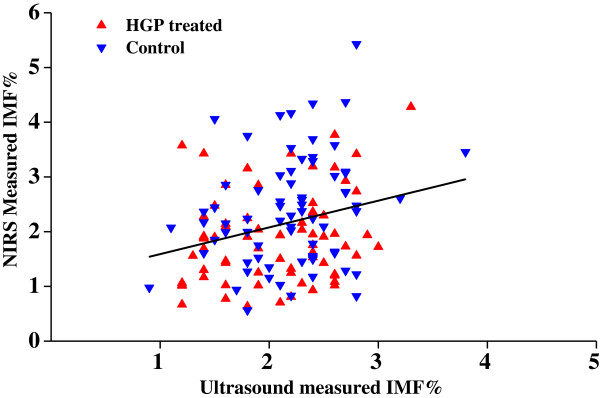
Correlation between ultrasound estimated IMF% and NIRS measured IMF% in Brahman cattle.

We have identified robust gene signals strongly correlated with IMF% in both cattle and sheep. We have also determined a simple linear relationship between IMF% and expression of the genes. How can we apply these gene expression assays in the cattle and sheep production industries to estimate IMF%? Below we discuss two example applications of the sets of genes for use in the cattle and sheep production industries: ranking animals by IMF% within a group (such as on a single farm), or comparing difference in IMF% between two groups (such as two different treatments or farms). Some methods described are only applicable within groups of animals, and the others can be applied in both situations. HGP-treatment is expected to reduce IMF% in both cattle and sheep [11,28]. This provides us with a good example to test the reliability of a gene, or gene combinations, to estimate IMF% in both species. We chose the WA HGP-treated and control groups for the example (Table 4). These animals are subsets of a larger group of 173 animals with the same treatments and from the same site [12,29]. We have shown above that at the time of sampling in this experiment HGP-treatment did not have a major effect on the relationship between IMF% and gene expression relative to the control group (Figure 2A&C). Thus the ability of the methods to differentiate between the two groups (with the HGP-treated group having a lower average measured or estimated IMF% or ranking) is a reflection of the strength of the relationship between the result of the method and the IMF% of individuals. There was no significant difference in average IMF% (NIRS measured) between the WA HGP-treated and WA control subgroups for which gene expression data was available (Table 4). As discussed previously [4], a reduction in IMF% caused by HGP-treatment would not be expected to be detectable in such small groups of animals. For the whole set of 141 WA animals (IMF% data was not available for 32 animals), HGP-treated and control animals could be significantly differentiated by directly measured IMF% (*P*-value = 0.001). But ultrasound estimated IMF% did not discriminate between the two groups, even when data from 173 animals was used (Table 4). Consistent with this IMF% estimated by ultrasound was not correlated with NIRS measured IMF%, R^2^ is 0.086 (Figure 4). These results were not unexpected as ultrasound is not recommended as a method for estimating IMF% in cattle with less than 2% IMF% [13]. Half of the Brahman steers from WA had NIRS measured IMF% of less than 2%. More typical use of ultrasound in Australia to estimate IMF% is in the range 2-8% where proficient scanners achieve correlation of ultrasound estimated IMF% with measured IMF% well in excess of 0.75 [13].

**Table 4:** Comparison of the performance of different measures and estimators of IMF%

**Method**	**Number of animals**^**1**^	**WA control subgroup**	**WA HGP subgroup**	***P*-value**^**2**^	**Predicted experiment size**^**3**^
NIRS measured IMF%	141	2.37 ± 1.00^4^	1.90 ± 0.83	0.001	198
Ultrasound estimated IMF%	173	2.66 ± 0.72^4^	2.93 ± 0.54	1.000	N/A^5^
NIRS measured IMF%	22	2.07 ± 0.77^4^	1.79 ± 0.54	0.340	294
IMF% calculated by *CIDEA* formula	22	2.30 ± 1.19^6^	1.60 ± 1.14	0.080	148
IMF% calculated by IMF 5 gene set formula	22	2.66 ± 1.04^6^	1.30 ± 0.92	0.003	26
Ranking animals using *CIDEA*	22	9.50 ± 6.62^7^	13.20 ± 6.16	0.100	156
Ranking animals using IMF 5 gene set	22	7.20 ± 5.47^8^	15.10 ± 4.99	0.0026	22
Ranking animals using TAG gene set	22	7.00 ± 4.52^8^	15.30 ± 5.48	0.0017	20
*CIDEA* DE	22	13.09 ± 0.44^7^	12.83 ± 0.38	0.080	130
IMF 5 gene set DE	22	0.25 ± 0.38^9^	−0.27 ± 0.31	0.0014	24
TAG gene set DE	22	0.28 ± 0.36^9^	−0.27 ± 0.33	0.00076	20

^1^Number of animals used for the analysis.

^2^For the test that average measured or estimated IMF%/gene expression/ranking in HGP-treated animals is lower than in control animals.

^3^The sample size predicted to be required to observe a significant result (P < 0.05) with 95% confidence intervals.

^4^Mean values, standard deviation and *P*-values are calculated using original record data.

^5^Ultrasound estimated IMF% did not detect an effect of the expected direction.

^6^Mean values, standard deviation and *P*-values are calculated using rescaled gene expression values.

^7^Mean values, standard deviation and *P*-values of ranking and DE of *CIDEA* are calculated using rescaled gene expression values.

^8^Mean values, standard deviation and *P*-values of ranking calculation of IMF 5 gene set and TAG gene set are based on Mann-Witney test.

^9^Mean values, standard deviation and *P*-values of IMF 5 gene set and TAG gene set DE are calculated using rescaled gene expression values.

To demonstrate the two applications of our findings, the regression equations based on the relationship between IMF% and expression of *CIDEA*, or the IMF 5 gene set, were used to estimate the IMF% for each animal. For both approaches there was a significant difference between the means of the estimated IMF% of the two groups (*P <* 0.05), unlike for the NIRS measured IMF% on the same 22 animals (*P =* 0.34) (Table 4). Although the correlation between *CIDEA* expression and IMF%, and the expression of the IMF 5 gene set with IMF% were very similar (Table 3). The use of more genes appears to significantly improve the accuracy of the estimation of IMF% (Table 4).

Rather than calculating an estimated IMF%, animals can be ranked on the basis of the relative gene expression values. The results showed that there was a significant difference between the average rankings of animals in the WA HGP-treated and control groups using the IMF 5 gene set (*P*-value = 0.0026) and TAG gene set (*P*-value = 0.0017), but not using *CIDEA* alone (*P*-value = 0.1) (Table 4). Again the apparent accuracy of the ranking method was improved significantly by the inclusion of additional genes, although increasing to 15 genes provided little additional improvement.

To compare IMF% of animals between different farms, besides the approaches above, we could also compare the DE of *CIDEA*, IMF gene set or TAG gene sets between two groups of animals. Rescaled gene expression data was used for the DE calculation of the multiple genes in the IMF 5 gene set and TAG gene set. As in the ranking method above, increasing the number of genes from one to five increased the discrimination between the two groups, but increasing the number of genes to 15 had little further effect (Table 4).

Generally speaking, in both research and production settings, the number of animals tested is the major determinant of the cost of collecting phenotypes. Methods which reduce the number of animals required to be tested and/or which can be conducted without sacrificing the animal will reduce the costs of phenotyping substantially. Using the approach of LaMorte [30], we estimated the sample size required to detect an affect at *P* < 0.05 and a confidence interval of >95% for all of the analysis methods (Table 4). Given the small size of the datasets the sample sizes for approaches using gene expression are likely to be overestimated. No reliable estimate of the sample size could be made for the use of ultrasound as the available data confirms that ultrasound is inaccurate for animals with low IMF%. To detect the effect using NIRS measured IMF% a large number of animals are required (Table 4). Using gene expression of *CIDEA* a slightly smaller sample size may be adequate. However, the use of five genes substantially reduced the predicted sample size, suggesting that around one eighth of the number of animals may be required to detect the effect of HGPs on IMF%. This improvement in performance and hence reduction in experiment size may be because the use of a multiple gene set effectively provides multiple measurements of the phenotype (deposition of TAG in lipid droplets in intramuscular adipocytes) leading to a reduced measurement error than using *CIDEA* alone, or the mean of duplicate NIRS measurements.

**Figure 5:**
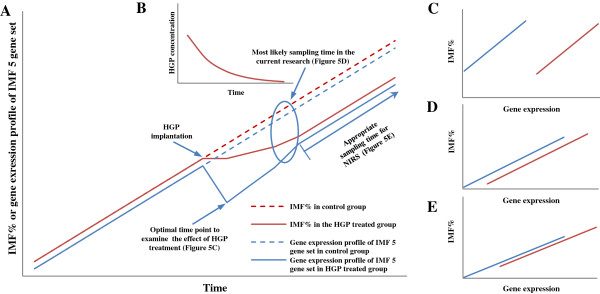
**Diagrammatic relationship of measured IMF% and gene expression of IMF 5 gene set over time. A)** Linear parallel portions of the curves for IMF% and gene expression have been separated for display purposes only. **B)** Profile of the concentration of circulating HGP after implantation. **C), D) and E)** Relationship between IMF% and gene expression of IMF 5 gene set at different time points after HGP implantation. The red solid lines represented control subgroup; blue ones represented the HGP treated subgroup.

The estimation of IMF% using gene expression is successful probably because gene expression of the IMF 5 gene set is proportional to IMF deposition rate and in growing animals depositing IMF, and yet to reach maturity, IMF deposition rate is proportional to IMF%.However, the improvement in performance of the gene expression over NIRS to identify the inhibitory effect of HGP-treatment may also be partly due to the following model. Within a short time after HGP treatment, intramuscular fat deposition may almost stop and as a consequence the expression of the IMF gene set genes would be predicted to be greatly reduced (Figure 5A). The concentration of circulating HGP would decrease with time (Figure 5B), so the effect of HGP on intramuscular fat deposition processes and the expression of the IMF gene set would be predicted to be gradually reduced and finally recover to the normal state described above (Figure 5A). If this model is correct, then shortly after HGP treatment, there would be little difference in measured IMF%, but a large difference in gene expression, between the treated and the control animals. At this point, gene expression would have a much larger discriminating power than IMF measurement (Figure 5A, C). Subsequently, the differences between measured IMF% and estimated IMF% based on gene expression would decrease as the concentration of circulating HGP reduces. During this period, gene expression would still be predicted to have more discriminating power than the direct measurement of IMF%. Eventually, the increased power of gene expression would be reduced to that normally observed (Figure 5A, E). The exact location on this theoretical curve of the experimental animals used in this work is unknown, but may be whilst there is still some additional discriminating power due to the effect described above (Figure 5A, D). However, certainly the animals were not at the optimal discrimination point (Figures 2A, C, 5A, C). Whilst it is likely that fewer animals were required for discrimination between HGP-treated and control animals using gene expression than were required for NIRS measured IMF% due to the effect described above and the intrinsic nature of the assay, the relative contributions of the two factors is unclear.

## Conclusion

By integrating data from cattle and sheep we have identified a set of 30 genes with robust correlation with IMF% in both cattle and sheep LM. Based on this gene set, we identified *CIDEA* as the gene whose expression was most correlated with IMF% in both cattle and sheep. Whilst *CIDEA* alone could be used to estimate IMF%, it is of similar utility to NIRS measured IMF%. In contrast, the non-invasive technique of ultrasound did not perform adequately on animals with low IMF%. By combining the data from 5 genes apparently improved estimates of IMF% could be calculated, with a commensurate reduction in the experiment size required to detect the impact of a treatment on IMF%. The five gene set can be used to estimate IMF% (based on the proposed relationship between the expression of the IMF 5 gene set, IMF deposition rate and IMF%) from biopsy as well as post slaughter samples, and on samples from animals with low IMF%, such as the Brahmans used in this work and younger animals of higher marbling breeds. The approach to phenotyping animals using gene expression shows promise as an alternative to current approaches for the measurement/estimation of IMF% in both cattle and sheep.

In addition, we have described a potentially generic approach to the development of robust gene expression phenotypes for other phenotypes of industry importance. The pipeline is as follows: calculate the correlation between gene expression and a phenotype (IMF% in this paper) and/or DE in two or more different groups of animals with significantly different experimental structures and phenotypic performance to generate the corresponding datasets. Then rank the genes based on the coefficients above in each dataset to primarily select a group of genes highly correlated with this phenotype and with each other across the different groups of animals. Lastly, optimise this group of genes based on their biological function to identify a gene set with appropriate size.

## Competing interests

The authors declare no competing interest regarding the content or conclusions expressed in this research.

## Authors’ contributions

The experimental design was mainly conceived by BPD. Samples and data were generated by KK and PLG. Data analysis was carried out by BG under the guidance of BPD. GZ, WZ and BPD resourced the project. BG and BPD were the primary authors of the paper, but all the authors contributed to, read and approved the final manuscript.

## Supplementary Material

Additional file 1: Table S1 Clustering parameters, correlation/DE coefficients and rankings of genes for use in cluster analysis. Click here for file
